# Glycyrrhizin Ameliorates Learning and Memory Impairment via Inhibition of Neuroinflammation in an Alzheimer’s Disease Mouse Model SAMP8

**DOI:** 10.3390/ijms27167399

**Published:** 2026-08-19

**Authors:** Guifeng Wang, Keiichi Hiramoto, Ning Ma, Shiho Ohnishi, Nobuji Yoshikawa, Mariko Murata, Shosuke Kawanishi

**Affiliations:** 1 Department of Acupuncture and Moxibustion Medical Science, Suzuka University of Medical Science, Suzuka 510-0293, Mie, Japan; wang@suzuka-u.ac.jp; 2 Faculty of Pharmaceutical Sciences, Suzuka University of Medical Science, Suzuka 513-8670, Mie, Japan; hiramoto@suzuka-u.ac.jp (K.H.);; 3 Graduate School of Health Science, Suzuka University of Medical Science, Suzuka 510-0293, Mie, Japan; maning@suzuka-u.ac.jp; 4 Institute of Traditional Chinese Medicine, Suzuka University of Medical Science, Suzuka 510-0293, Mie, Japan; 5 Matsusaka R&D Center, Cokey Co., Ltd., Matsusaka 515-0041, Mie, Japan; yoshikawa@cokey.co.jp; 6 Department of Environmental and Molecular Medicine, Mie University Graduate School of Medicine, Tsu 514-8507, Mie, Japan

**Keywords:** glycyrrhizin, phosphorylated tau, neuroinflammation, α-Klotho, 2′,3′-cGAMP, HMGB1

## Abstract

Neuroinflammation plays a central role in Alzheimer’s disease (AD). Glycyrrhizin (GL), a major component of licorice, exhibits anti-inflammatory effects, but its effects on AD pathology remain unclear. To investigate the effects of GL (18β-glycyrrhizin, 18β-GL) and its stereoisomer (18α-glycyrrhizin, 18α-GL) on cognitive function, neuroinflammation, and AD pathology in senescence-accelerated mouse prone 8 (SAMP8; P8) mice, 40-week-old P8 male mice, an AD model due to aging, and the control (senescence-accelerated mouse resistant 1, SAMR1; R1) mice were treated with 18β-GL, 18α-GL and physiological saline (control) for 12 weeks (n = 6 in each group). Cognitive function was evaluated using a step-through passive avoidance test. Plasma levels of α-Klotho, IGF-1, 2′,3′-cyclic GMP-AMP (2′,3′-cGAMP), HMGB1, IL-6, and TNF-α were measured by ELISA. Hippocampal microglial activation (Iba1), amyloid-β (Aβ) deposition, and phosphorylated tau (p-Tau) were assessed by immunohistochemistry. Aged P8 mice showed impaired memory, decreased α-Klotho and IGF-1 levels, and increased inflammatory markers compared with R1 mice. GL significantly improved memory performance, reduced inflammatory markers, and suppressed Iba1 activation, as well as Aβ and p-Tau accumulation. These effects were associated with inhibition of the cGAS–STING pathway, as indicated by reduced 2′,3′-cGAMP and HMGB1 levels. GL ameliorates AD pathology by inhibiting neuroinflammation, suggesting its therapeutic potential for AD.

## 1. Introduction

Neuroinflammation accompanied by microglial activation is one of the primary mechanisms leading to neuronal death and dysfunction in neurodegenerative diseases including Alzheimer’s disease (AD) [1]. AD is the most common and well-known cause of dementia and is characterized by progressive deficits in memory and cognitive function, driven by amyloid-β (Aβ) and phosphorylated tau protein (p-Tau).

Various inflammation-related factors involved in the progression of AD. It is considered that α-Klotho and insulin-like growth factor I (IGF-1) are inhibitory factors against AD progression. α-Klotho, a longevity factor, declines with aging, and its peripheral administration improved cognitive function in aged mice [2]. α-Klotho has several beneficial effects against brain aging, including reduction in oxidative stress, modulation of inflammatory responses, and enhancement of synaptic plasticity, memory, and learning [3]. A recent review [4] highlights a potential protective role of the IGF-1 pathway in the pathogenesis of neurodegenerative diseases, including AD. On the other hand, we postulate several factors that accelerate the progression of AD. High mobility group box 1 (HMGB1) protein, an initiator and activator of neuroinflammatory responses, is implicated in the pathogenesis of AD [5]. In recent years, the cyclic GMP–AMP synthase (cGAS)–2′,3′-cGAMP–stimulator of interferon genes (STING) pathway, an immune response crucial for intracellular DNA sensing, has increasingly garnered attention for its role in neurodegenerative disorders [6].

Cognitive impairment, memory loss, and other neurodegenerative disorders are frequently observed over the long term in the elderly. The onset of sporadic Alzheimer’s disease is considered to be age-dependent. Senescence-accelerated mouse prone 8 (SAMP8) mice have been established as a model for accelerated brain aging and the neuropathology of dementia, and their phenotype resembles that of patients with late-onset and age-related sporadic AD [7,8]. Transgenic mouse models, such as 5XFAD and 3xTg mice, are the most common models used to mimic familial AD, but these mutations account for only a small fraction of clinical cases. The SAMP8 strain is an animal model that spontaneously exhibits accelerated aging. SAMP8 mice serve as a powerful model for elucidating the etiology and pathogenesis of sporadic AD and are considered highly valid experimental models for developing preventive and therapeutic strategies for late-onset (age-related) AD, which accounts for the majority of AD cases.

Glycyrrhizin (GL) is the principal component of licorice root, which is used as a Chinese herbal medicine (Kakkonto, Shosaikoto, etc.) for its anti-inflammatory properties and as a sweetener. Although GL consists primarily of 18β-glycyrrhizinic acid (18β-GL), another stereoisomer, 18α-glycyrrhizinic acid (18α-GL), also exists. The chemical structure name for 18β-GL is [(18β)-20β-carboxy-11-oxo-30-norolean-12-en-3β-yl]2-O-β-D-glucopyranuronosyl-α-D-glucopyranosiduronic acid. Also, 18α-GL is [(18α)-20β-carboxy-11-oxo-30-norolean-12-en-3β-yl]2-O-β-D-glucopyranuronosyl-α-D-glucopyranosiduronic acid. Interestingly, Amagaya et al. [9] conducted a comparative study using the aglycone metabolites of 18β-GL and 18α-GL, namely, 18β- and 18α-glycyrrhetinic acid (GA), and demonstrated that 18α-GA exhibited greater anti-inflammatory activity than 18β-GA. It has been reported that 18α-GA acts as a proteasome activator in cell cultures, enhancing proteasome activity levels, accompanied by a reduction in Aβ deposits, and ultimately slowing the progression of AD phenotype [10]. In contrast, an in vitro study showed that 18β-GA exhibited a higher inhibitory capacity against beta-site amyloid precursor protein cleaving enzyme 1 (BACE1) than 18α-GA [11]. The effects of the GL stereoisomers on AD remain unclear.

This study focused on neuroinflammation and AD associated with aging, aiming to elucidate the effects of GL on AD. We investigated the effects of both 18β-GL and 18α-GL on learning and memory function of SAMP8 (P8) mice and their normal control (senescence-accelerated mouse resistant 1, SAMR1; R1) mice, and analyzed blood-based inflammation-related factors using ELISA and AD pathogenesis-related factors, such as activated microglia, Aβ, and p-Tau protein in the hippocampus using immunohistochemistry (IHC).

## 2. Results

### 2.1. Effects of GL on Learning and Memory Performance in an AD Mouse Model

**Figure 1:**
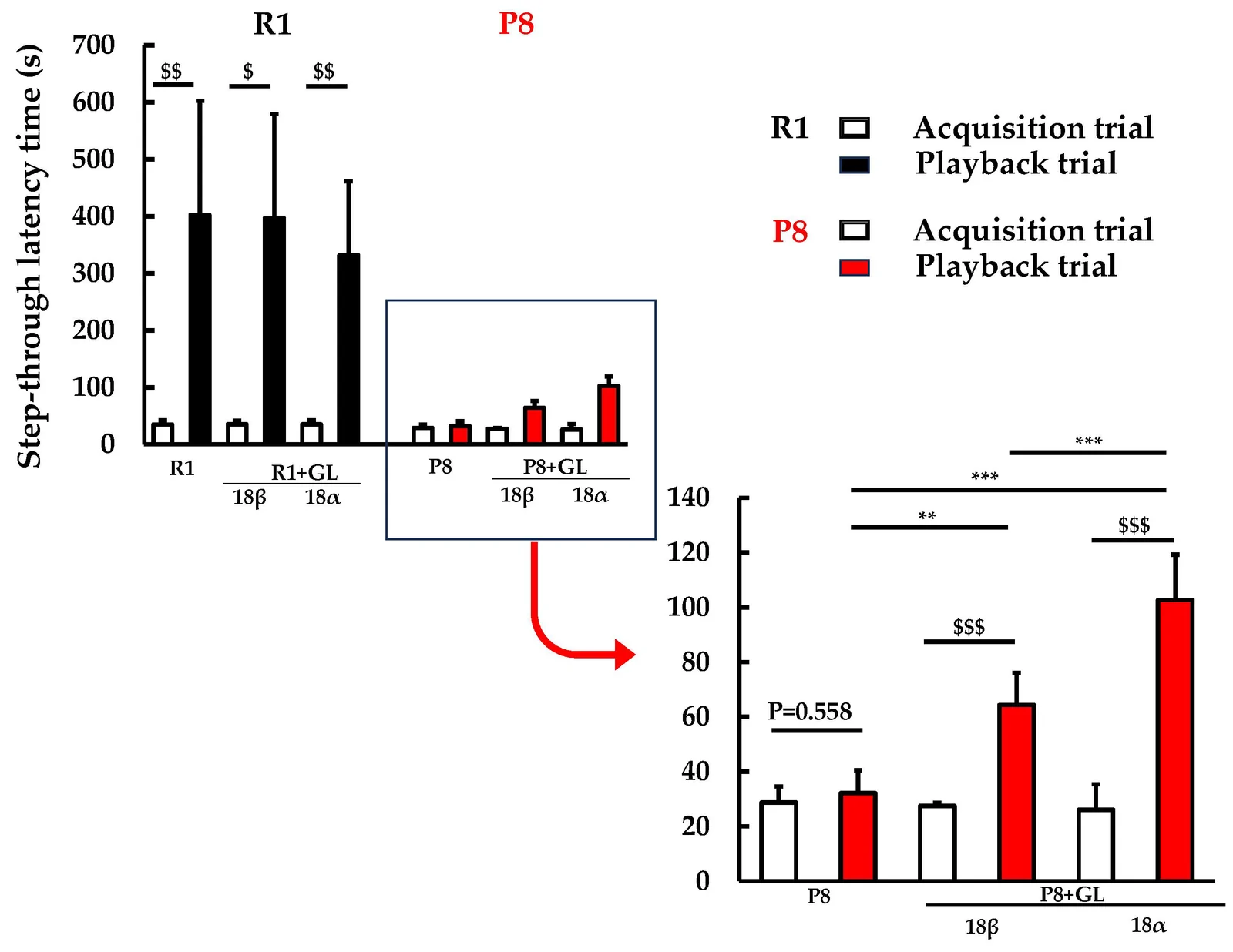
Effects of GL isomers on passive avoidance performance. Step-through latency was measured during acquisition and playback trials in R1 and P8 groups with or without 18β-GL and its 18α isomer. Data are presented as mean ± SD. $; *p* < 0.05, $$; *p* < 0.01, $$$; *p* < 0.001 by paired *t* test. **; *p* < 0.01, ***; *p* < 0.001 by ANOVA with Tukey’s HSD test.

In the learning and memory test (step-through passive avoidance test) (Figure 1), R1 mice showed a significant increase in latency to enter the shock-associated compartment under all treatment conditions (*p* < 0.05 or *p* < 0.01), indicating normal learning ability. In contrast, P8 control mice did not show a significant increase in latency between acquisition trial and playback trial (*p* = 0.558), suggesting that learning and memory function were impaired. However, administration of GL significantly improved the latency in P8 mice (*p* < 0.001), indicating an improvement in learning and memory performance. In addition, the latency of the playback trial was increased in GL groups compared to the control P8 group (*p* < 0.01), and the increase was greater in the 18α-GL group than that in the 18β-GL group (*p* < 0.01). These results suggest that GL, especially 18α-GL, may ameliorate learning and memory impairment in SAMP8 mice.

### 2.2. Effects of GL on Plasma Levels of α-Klotho, IGF-1, 2′,3′-cGAMP, HMGB1, IL-6, and TNF-α in an AD Mouse Model

**Figure 2:**
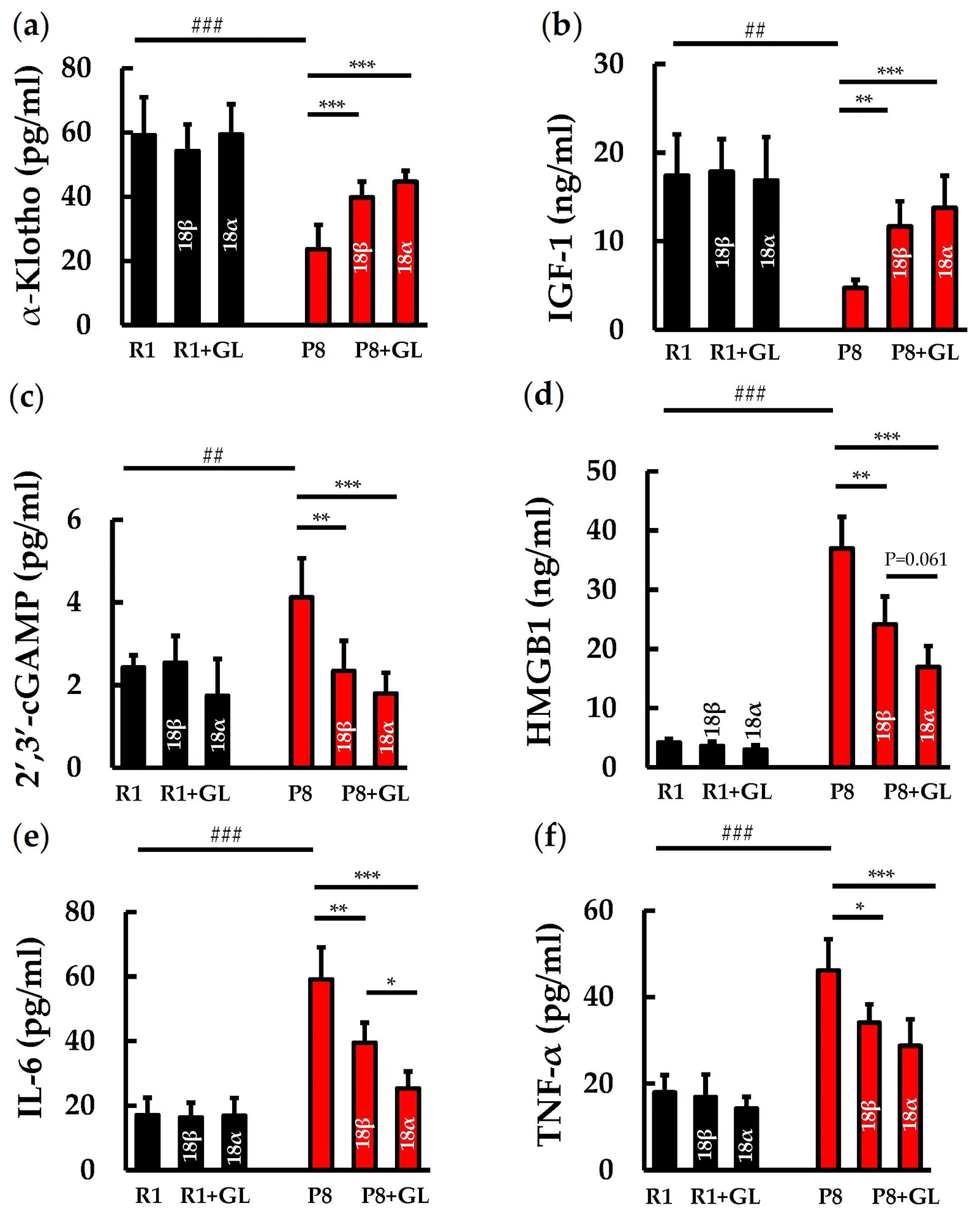
Effects of GL on plasma levels of α-Klotho, IGF-1, 2′,3′-cGAMP, HMGB1, IL-6, and TNF-α in an AD mouse model. Plasma levels of (**a**) α-Klotho, (**b**) IGF-1, (**c**) 2′,3′-cGAMP, (**d**) HMGB1, (**e**) IL-6, and (**f**) TNF-α were measured in R1 and P8 groups with or without 18β-GL and its 18α isomer. Data are presented as mean ± SD. ##; *p* < 0.01, ###; *p* < 0.001 by Student’s *t* test. *; *p* < 0.05, **; *p* < 0.01, ***; *p* < 0.001 by ANOVA with Tukey’s HSD test.

In blood samples from SAM mice, the plasma α-Klotho level (Figure 2a) was significantly lower in the P8 control group than in the R1 control group (*p* < 0.001). In the P8 groups, GL administration significantly increased the α-Klotho level (*p* < 0.001). The plasma level of IGF-1, a neurotrophic factor (Figure 2b), was significantly lower in the P8 control group than in the R1 control group (*p* < 0.01). In the P8 groups, GL administration significantly increased IGF-1 levels compared with the control group (18β-GL, *p* < 0.01; 18α-GL, *p* < 0.001). These results indicate that the senescence-related decreases in α-Klotho and IGF-1 levels were ameliorated by GL administration.

The plasma level of 2′,3′-cGAMP, an important second messenger from the cytosolic DNA sensor cGAS–STING pathway (Figure 2c), was significantly increased in the P8 control group compared with the R1 control group (*p* < 0.01). In the P8 groups, GL administration significantly decreased 2′,3′-cGAMP levels compared with the control group (18β-GL, *p* < 0.01; 18α-GL, *p* < 0.001). The plasma level of HMGB1, an inflammatory mediator and also an extracellular stress marker (Figure 2d), was significantly elevated in the P8 control group compared with the R1 control group (*p* < 0.001). In the P8 groups, GL administration significantly reduced HMGB1 levels compared with the control group (18β-GL, *p* < 0.01; 18α-GL, *p* < 0.001), and 18α-GL treatment tended to decrease HMGB1 more than 18β-GL treatment (*p* = 0.061). These results suggest that the activation of the HMGB1-cGAS-STING axis may be suppressed by GL administration.

The plasma levels of the inflammatory cytokines IL-6 and TNF-α (Figure 2e and Figure 2f, respectively) were significantly higher in the P8 control group than in the R1 control group (*p* < 0.001). GL administration significantly reduced the levels of these cytokines. In the P8 groups, IL-6 level (Figure 2e) was significantly decreased in the 18β-GL group (*p* < 0.01) and 18α-GL group (*p* < 0.001) compared with the control group, and the 18α-GL treatment showed a significantly greater reduction than the 18β-GL treatment (*p* < 0.05). In the P8 groups, TNF-α level (Figure 2f) was significantly decreased in the 18β-GL group (*p* < 0.05) and the 18α-GL group (*p* < 0.001) compared with the control group. These results suggest that the increases in inflammatory cytokines are suppressed by GL, especially 18α-GL, administration.

### 2.3. Effects of GL on Iba1 Expression in the Hippocampus of AD Model Mice

**Figure 3:**
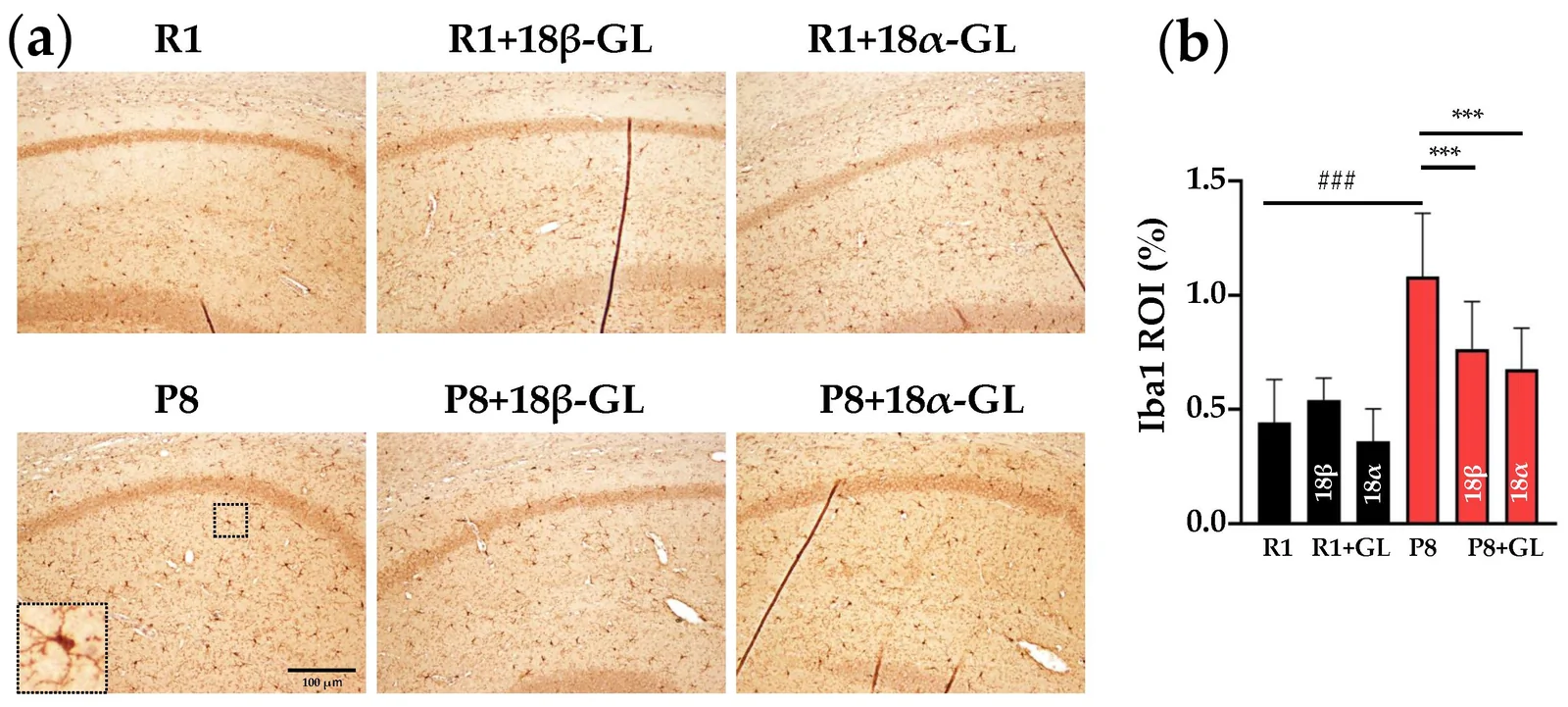
Effects of GL on Iba1 expression in the hippocampus of AD model mice. (**a**) Representative IHC images of Iba1 expression in the hippocampus of R1 and P8 groups with or without 18β-GL and its 18α isomer. Insets show higher magnification views of the indicated regions. Scale bar = 100 μm. (**b**) Quantification of Iba1-positive area (ROI%). Data are presented as mean ± SD. ###; *p* < 0.001 by Student’s *t* test. ***; *p* < 0.001 by ANOVA with Tukey’s HSD test.

IHC analysis was performed to evaluate the expression of the microglial marker Iba1 in hippocampal tissue sections from SAM mouse brains (Figure 3a). Many of the Iba1-positive cells in the P8 control group were enlarged, and their number increased, which is consistent with the features of activated microglia [12,13]. Compared with the R1 control group, the ROI (%) of Iba1-positive staining area (Figure 3b) was significantly increased in the P8 control group (*p* < 0.001). In the P8 groups, GL administration significantly reduced the Iba1-positive ROI (%) (18β-GL, 18α-GL: *p* < 0.001). These results suggest that GL may suppress microglial activation in AD model mice.

### 2.4. Effects of GL on Aβ Deposition in the Hippocampus of AD Model Mice

**Figure 4:**
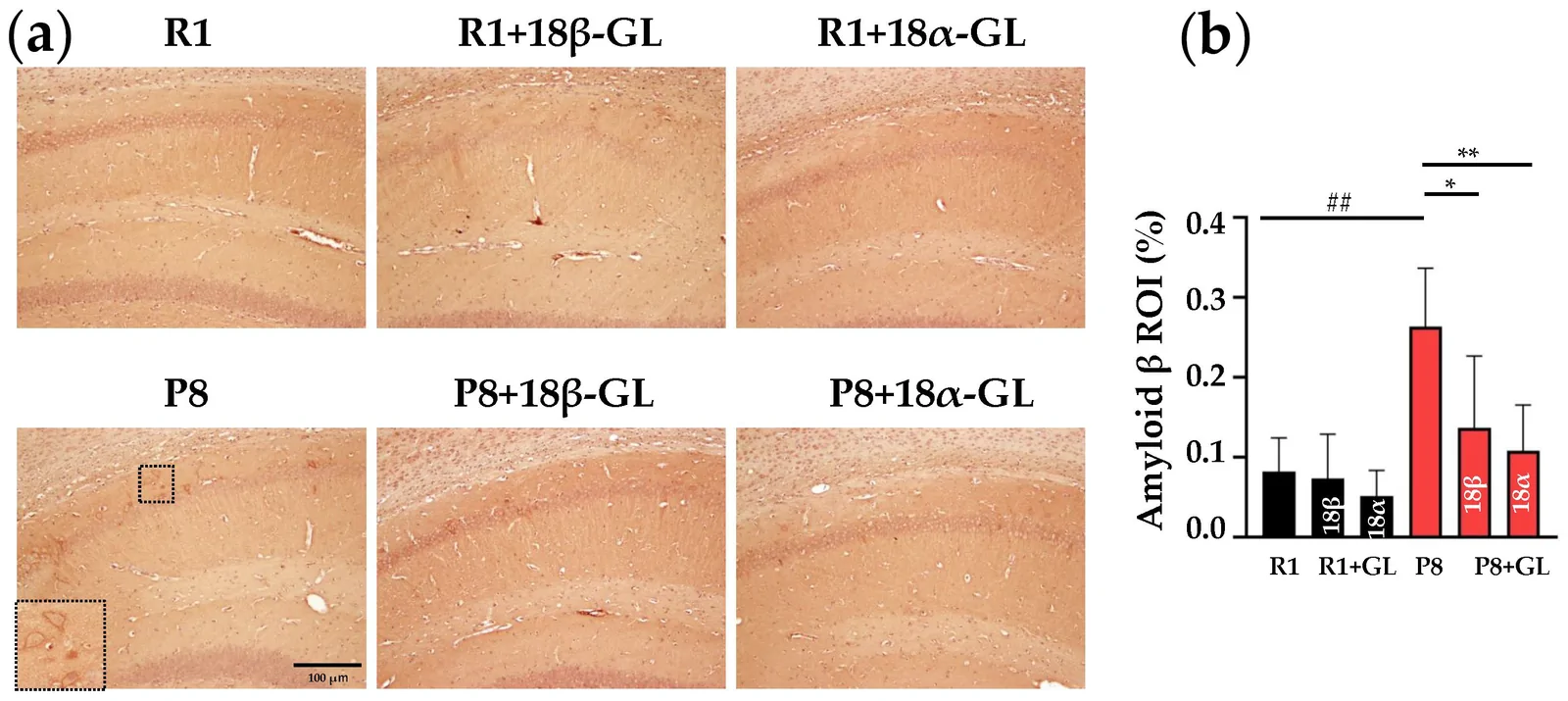
Effects of GL on Aβ deposition accumulation in the hippocampus of AD model mice. (**a**) Representative IHC images of Aβ deposition in the hippocampus of R1 and P8 groups with or without GL isomers (18β-GL and 18α-GL). Insets show higher-magnification views of the indicated regions. Scale bar = 100 μm. (**b**) Quantitative analysis (ROI%) of Aβ-positive area. Data are presented as mean ± SD. ##; *p* < 0.01 by Student’s *t* test. *; *p* < 0.05, **; *p* < 0.01 by ANOVA with Tukey’s HSD test.

Aβ deposition was analyzed immunohistochemically in hippocampal tissue sections from SAMP mouse brains (Figure 4a). Compared with the R1 control group, the IHC score of Aβ was significantly higher in the P8 control group (*p* < 0.01). Compared with the R1 control group, the ROI (%) (Figure 4b) of Aβ was significantly higher in the P8 control group (*p* < 0.01). In the P8 groups, the ROI (%) was significantly reduced in the GL groups compared with the control group (18β-GL, *p* < 0.05; 18α-GL, *p* < 0.01). These results suggest that GLs can suppress Aβ accumulation in AD model mice.

### 2.5. Effects of GL on p-Tau Deposition in the Hippocampus of AD Model Mice

**Figure 5:**
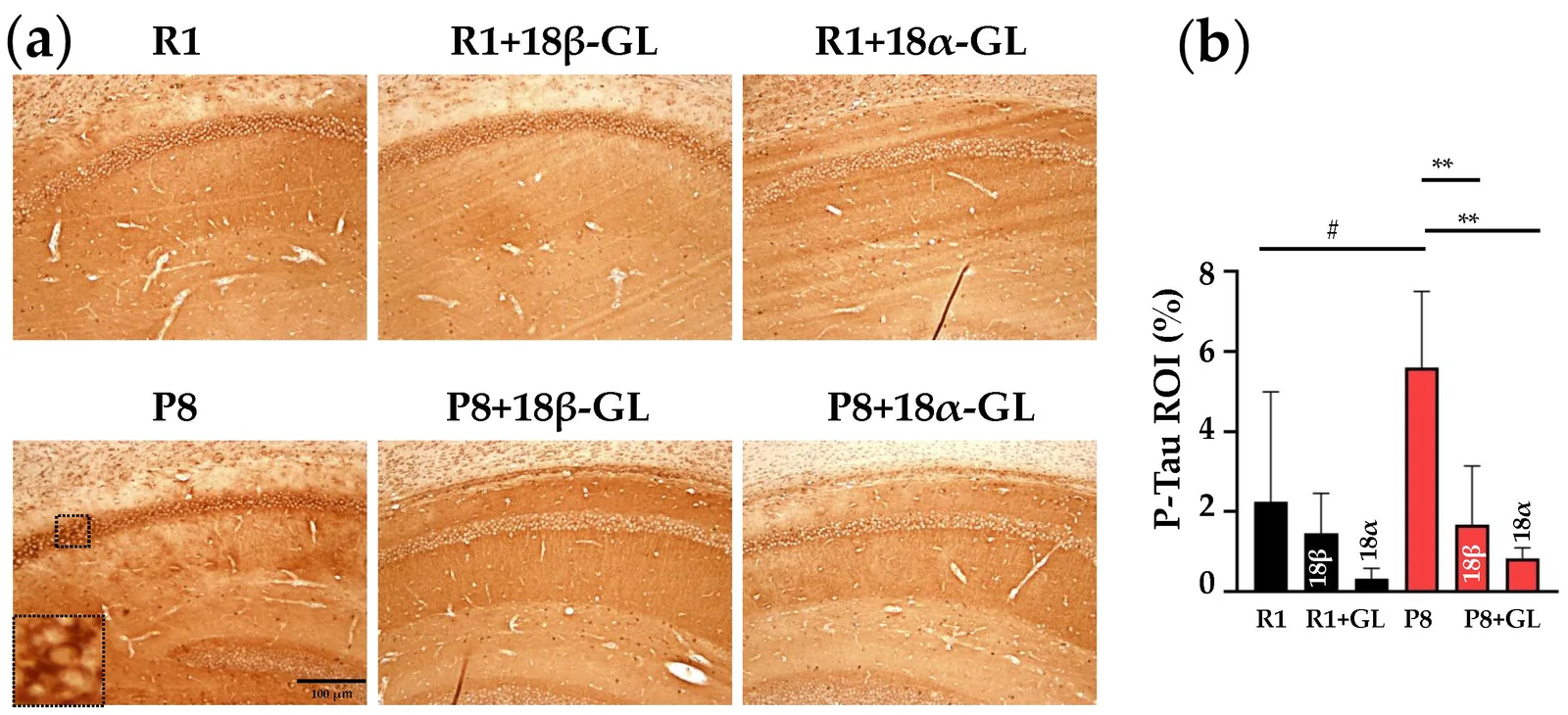
Effects of GL on p-Tau accumulation in the hippocampus of AD model mice. (**a**) Representative IHC images of p-Tau accumulation in the hippocampus of R1 and P8 groups with or without GL isomers (18β-GL and 18α-GL). Insets show higher magnification views of the indicated regions. Scale bar = 100 μm. (**b**) Quantitative analysis (ROI%) of p-Tau -positive area. Data are presented as mean ± SD. #; *p* < 0.05 by Student’s *t* test. **; *p* < 0.01 by ANOVA with Tukey’s HSD test.

The accumulation of p-Tau protein was analyzed immunohistochemically in hippocampal tissue sections from SAM mouse brains (Figure 5a). Compared with the R1 control group, the ROI (%) (Figure 5b) of p-Tau was significantly higher in the P8 control group (*p* < 0.05). In the P8 groups, the ROI (%) was significantly reduced in the GL groups compared with the control group (*p* < 0.01). These results suggest that GLs suppress the phosphorylation of tau protein in AD model mice.

## 3. Discussion

In this study, we demonstrated that SAMP8 mice exhibited learning and memory impairments compared to the control R1 mice. These impairments were accompanied by heightened microglial activation and the accumulation of Aβ and p-Tau in the hippocampus, with a reduction in circulating levels of the anti-aging factor α-Klotho and the neurotrophic factor IGF-1, and an increase in inflammation-related factors (2′,3′-cGAMP, HMGB1, IL-6, and TNF-α). GL ameliorated these AD-associated changes and inflammation. Notably, 18α-GL demonstrated significantly superior efficacy compared to 18β-GL in improving learning and memory functions and reducing plasma IL-6 concentrations. Elevated concentrations of IL-6 [14] and HMGB1 [15] have been reported in the blood of AD patients compared to controls. Efforts are currently underway to identify and validate innovative blood-based biomarkers that effectively reflect AD-related pathological mechanisms at a peripheral level [16]. A recent review [17] suggests that neuroinflammation, including cGAS-STING signaling, affects blood–brain barrier (BBB) integrity, and that dysregulated activation of this pathway promotes barrier breakdown. One plausible mechanism is that BBB dysfunction resulting from neuroinflammation influences the blood concentrations of the molecules observed in this study. Future research is needed to elucidate the relationship between the expression levels of these molecules in the brain and in the blood.

**Figure 6:**
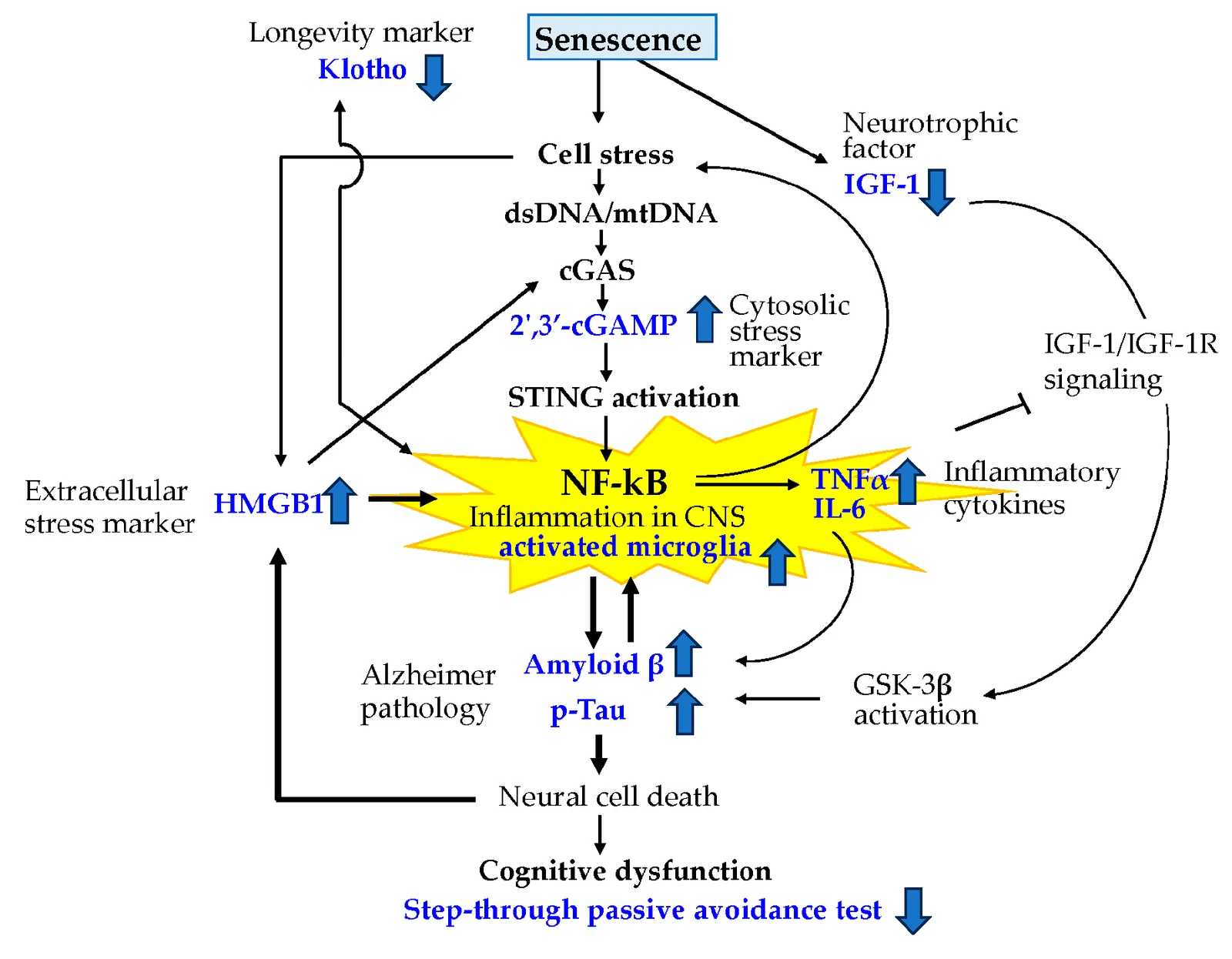
Proposed mechanism of AD pathology. Aging-related cellular stress induces the release of dsDNA/mtDNA, leading to activation of the cGAS–STING pathway and increased production of 2′,3′-cGAMP. This promotes NF-κB activation and subsequent neuroinflammation, characterized by elevated inflammatory cytokines (e.g., TNF-α and IL-6) and microglial activation. Senescence-induced cell stress triggers HMGB1 release and inflammatory responses, and modulates α-Klotho and IGF-1 levels. The neuroinflammation contributes to Aβ deposition and tau phosphorylation, ultimately leading to neuronal damage and cognitive dysfunction.

Based on our results and the previous literature, we propose a potential mechanism of aging-related AD (Figure 6), in which GL ameliorates memory function and AD pathology by suppressing inflammation. Many age-related diseases, including AD, share the common characteristic of involving chronic, low-grade neuroinflammatory processes.

The α-Klotho protein is well known for its anti-aging properties, which include the suppression of oxidative stress and neuroinflammation. It is important to note that Klotho expression levels decline with age, and this reduction is associated with a decline in cognitive function [18,19]. Cui et al. [20] reported that serum levels of α-Klotho protein and IGF-1 in patients with mild cognitive impairment (MCI) were lower than those in age- and sex-matched controls, suggesting that higher levels of serum α-Klotho and IGF-1 serve as protective factors against cognitive impairment in MCI patients. Zhang et al. [21] suggested that α-Klotho enhances IGF-1 activity. IGF-1 exerts its physiological effects by binding to the IGF-1 receptor (IGF-1R) and activating the PI3K/Akt pathway. Chronic inflammation could suppress IGF-1 levels [22]. Dysregulation of IGF-1 signaling leads to the activation of GSK-3β, resulting in the accumulation of Aβ and p-Tau, and neural cell death [4]. A community-based study of middle-aged participants has shown that low serum IGF-1 levels are associated with an increased risk of developing AD and with brain atrophy [23]. In a rat model of AD, IGF-1 administration suppressed neuroinflammation and Aβ deposition in the hippocampus [24]. In the AD brain, the phosphatidylinositol 3-kinase (PI3K)/protein kinase B (Akt) neuroprotective pathway, which is involved in neuroprotection, is markedly inhibited, while glycogen synthase kinase 3 beta (GSK3β) is hyperactivated, and these alterations drive neurodegeneration through the hyperphosphorylation of tau protein [25,26]. Our results and the studies indicate that α-Klotho and IGF-1 are the protective factors against AD progression, and we are the first to report here the possibility of improving these factors by GL.

Senescence induces cell stress, and senescent cells release double-stranded DNA (dsDNA)/mitochondrial DNA (mtDNA) into the cytoplasm [27]. Cytosolic DNA is rapidly sensed by cGAS, which catalyzes the synthesis of 2′,3′-cGAMP. The second messenger 2′,3′-cGAMP binds to and activates STING, triggering NF-κB/IRF3 signaling that leads to the production of inflammatory cytokines and interferons [28,29,30]. In certain cancers, cell stress causes the release of complexes comprising HMGB1 and genomic DNA, which stimulate the cGAS-STING pathway [31,32,33,34]. These signals activate microglial cells and induce NF-κB, resulting in the release of cytokine mediators such as TNF-α and IL-6. Quintanilla et al. reported that IL-6 induced Alzheimer-type phosphorylation of tau protein by causing dysregulation of the cdk5/p35 pathway [35]. The overproduction of these mediators may trigger signaling cascades in neurons, potentially leading to tau hyperphosphorylation and the formation of neurotoxic oligomeric species through self-aggregation [36]. Due to senescence-induced cell stress and cell death, cells release HMGB1extracellularly, a protein known to be pro-inflammatory, into the extracellular space. This extracellular HMGB1 promotes inflammation in the pathology of numerous diseases, including cancer [37] and neurodegenerative disorders [38,39]. Extracellular HMGB1 binds to TLRs/RAGE and activates NF-κB signaling pathways, thereby inducing the proinflammatory cytokines TNF-α and IL-6. Recent studies using rat models of AD have demonstrated that HMGB1 gene therapy influences cognitive function and neuropathological characteristics [40]. Cytosolic and extracellular stress-induced inflammation in the central nervous system results in activation of microglia and accumulation of Aβ and p-Tau [41,42]. Enhanced NF-κB signaling increases the expression of BACE1 (beta-site APP cleaving enzyme 1), the enzyme responsible for cleaving the amyloid precursor protein (APP) to produce Aβ [43,44]. Concurrently, the deposition of Aβ and p-Tau oligomers induces cell stress, triggering the release of dsDNA and/or HMGB1 [41,45]. Aβ species activate microglia to secrete cytokines, which in turn regulate tau kinase [46]. Excessive Aβ deposition leads to the formation of senile plaques and hyperphosphorylation of tau protein, resulting in neurofibrillary tangles [47,48]. Furthermore, neuroinflammation accelerates Aβ deposition and p-Tau accumulation, creating a vicious cycle that drives the progression of neurodegenerative diseases. We have demonstrated that the vicious cycle of inflammation is extremely important in the development of senescence-related cognitive impairment.

In the mechanisms mentioned above, we hypothesize that GA is the active metabolite of GL. Upon oral administration, GL is degraded by β-D-glucuronidase in the intestine, generating GA. GL is metabolized into GA through the action of gut microbiota and is subsequently reabsorbed from the intestinal wall to exert its pharmacological effects [49]. Interestingly, it has been reported that GA was detected in the plasma, brain, and cerebrospinal fluid of rats following the oral administration of “Yokukansan,” a Kampo medicine containing GL [50]. This suggests that GL is absorbed into the bloodstream as GA and subsequently crosses the blood–brain barrier to reach the brain. Therefore, GL could exhibit anti-inflammatory effects on AD pathology in the hippocampus. GL is known to exhibit high affinity for HMGB1 and possess potent anti-inflammatory properties through the inhibition of the HMGB1/cGAS-STING/NF-κB signaling pathway. Furthermore, other signaling pathways, such as PI3K/AKT/GSK3β, p38-MAPK, and p53, have been implicated in the pharmacological mechanisms of GL [51,52]. An in vitro study using neuroblastoma cells showed that oligomeric Aβ_42_ peptides induced significant protein alterations associated with AD pathology, and GL protected the neuronal cells [53].

18β-GL is known to induce pseudo-hyperaldosteronism via inhibition of 11beta-HSD2. However, no studies have been conducted regarding the enzyme-inhibitory activity of 18α-GL. Classen-Houben et al. [54] revealed that while both isomers inhibited mouse 11β-HSD2, 18α-GA did not inhibit human 11β-HSD2, whereas 18β-GA did. Therefore, 18α-GL is considered superior to 18β-GL, as it offers high therapeutic efficacy without the risk of inducing pseudoaldosteronism. Given that 11beta-HSD1 catalyzes the conversion of inactive 11-ketoglucocorticoids into their active 11beta-hydroxy forms, the selective inhibition of 11beta-HSD1 by 18α-GA is currently regarded as a promising therapeutic strategy for the treatment of metabolic diseases. In a comparative study, rats administered the β-isomer exhibited elevated urinary cortisol/cortisone ratios, a biomarker for pseudoaldosteronism, suggesting that 18α-GL is a safer drug than 18β-GL [55]. Taken together, these findings indicate that 18α-GL may offer certain advantages over 18β-GL regarding its impact on health. It is necessary to clarify the differences in the effects of the stereoisomers on AD pathology in greater detail.

To confirm the beneficial effects of GL on cognitive function, further learning and memory behavioral tests are required in future studies. Although GL components hold the potential for anti-inflammatory effects, several challenges must be overcome for clinical application. In particular, significant issues remain regarding bioavailability, compound standardization, and clinical efficacy. Rigorous and well-controlled clinical trials are essential to establish the efficacy of GL isomers as a disease-modifying therapy for AD.

## 4. Materials and Methods

### 4.1. Experimental Protocol

A total of 38-week-old SAMP8 (P8) male mice and SAMR1 (R1) mice were purchased from Japan SLC, Inc. (Hamamatsu, Japan) and acclimatized for more than one week. SAMP8 mice are widely used as an animal model of AD in relation to aging [7,8]. It is well known that women are more susceptible to developing AD than men [56]. To elucidate the sex differences in the development of AD, similar experiments using female mice are required in the future. All animals were provided food and water ad libitum and housed in an SPF facility under a 12 h light/dark cycle (light phase: 08:00–20:00; dark phase: 20:00–08:00), at a temperature of 23.0 ± 1.0 °C and a humidity of 50 ± 10%. Mice were monitored daily.

GL, 18β-GL, and its stereoisomer 18α-GL were purchased from Cokey Co., Ltd. (Tokyo, Japan), and Chia-tai Tianqing Pharmaceutical Co. (Nanjing, China), respectively. GL was administered via oral gavage at a dose of 15 mg/kg three times weekly, starting at 40 weeks of age (for a total treatment period of 12 weeks). The control group received physiological saline. Phosphate-buffered saline was used to dissolve GL because of pH control. The dosage used in this study (15 mg/kg body weight) was set to be sufficiently low relative to the toxicity threshold [57] and within the safety margin used when evaluating the efficacy of the drug [58,59] in in vivo studies using rodents, but lower doses are used in food and clinical settings. Although the stereoisomer 18α-GL has not been used in previous animal studies, urinary excretion of sodium, potassium and calcium was increased only after dosing with 18α-GA, suggesting that it is considerably more toxic than the 18β-GA and GL [60]. Therefore, further research is needed regarding the dosage of GL, particularly 18α-GL.

To examine learning and memory, a passive avoidance (step-through) task was performed during the last week, as described below. To avoid the influence of learning effects, only a single set of trials was conducted.

**Figure 7:**
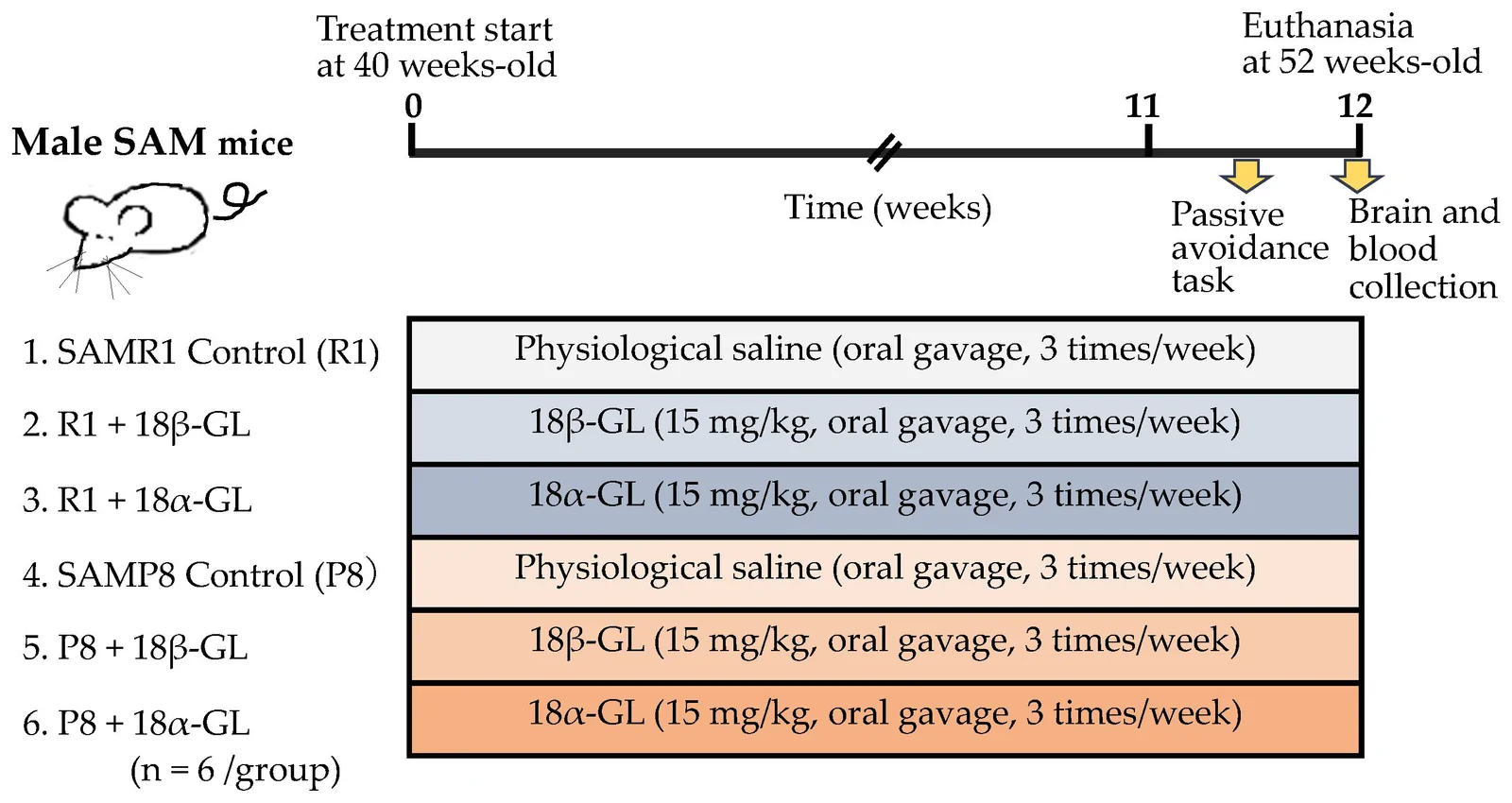
Experimental design and treatment protocol.

At the end of the experiment, mice were euthanized by overdose of sodium pentobarbital, and tissues including brain and blood were collected for subsequent analyses. The experimental protocol is shown in Figure 7. This experiment consists of six groups, with a sample size of six for each group.

All procedures were performed to minimize animal suffering during the experimental period. This study was conducted in accordance with the ARRIVE guidelines, and all experimental protocols were approved by the Animal Experimentation Committee of Suzuka University of Medical Science (Approval No. 89, 25 September 2025).

### 4.2. Learning and Memory Test: Step-Through Passive Avoidance Test

This test takes advantage of the mouse’s natural preference for dark environments. Briefly, each mouse was placed in the light compartment, and the latency to enter the dark compartment was recorded. Upon entering the dark compartment, the mouse received a mild foot shock for learning (acquisition trial). After 24 h, the mouse was again placed in the light compartment, and the latency to enter the dark compartment was measured (playback trial). If learning and memory were successfully formed, the mouse avoids the dark compartment, resulting in a prolonged latency.

### 4.3. Measurement of Plasma Levels of α-Klotho, IGF-1, 2′,3′-cGAMP, HMGB1, IL-6 and TNF-α

After blood collection, plasma was separated and analyzed using ELISA kits to measure α-Klotho (CUSABIO, Houston, TX, USA), IGF-1 (R&D Systems, Minneapolis, MN, USA), HMGB1 (Wuhan USCN Business Co., Ltd., Wuhan Economic & Technological Development Zone, Wuhan, China), 2′,3′-cGAMP (pg/mL) (Cayman Chemical, Ann Srbor, MI, USA), IL-6 (BioLegend, San Diego, CA, USA), and TNF-α (Proteintech, Rosemont, IL, USA).

### 4.4. Immunohistochemical Analysis

Immunohistochemical analyses were performed to examine the expression of Iba1, Aβ and p-Tau. The detailed procedures for immunohistochemical staining were carried out according to previously reported methods [61]. The primary antibodies used in this study were as follows: anti-Iba1 (Fujifilm Wako Pure Chemical Corporation, Okasa Japan, 013-27691, 1:500), anti-Tau FUJIFILM, RTM47, 1:500, and anti-β-amyloid (GeneTex, Irvine, CA USA, GT622, 1:200). Immunoreactivity was detected using an avidin–biotin complex method (Vectastain ABC kit, Vector Laboratories, Burlingame, CA, USA) with a DAB substrate kit (Nacalai Tesque Inc., Kyoto, Japan).

Stained brain sections were observed under an Olympus Bx51 light microscope with a DP-75 Digital camera. The number of Iba1-positive cells was counted. The immunopositive staining area was quantitatively analyzed by cellSens Dimension Software (version 4.3.1, Olympus, Tokyo, Japan). ROI (%) was defined as the percentage of immunopositive area relative to the total area within the region of interest.

### 4.5. Statistical Analysis

Statistical analyses were performed using an SPSS software (version 29.0 for Mac). We used analysis of variance (ANOVA) followed by Tukey’s multiple comparison test among three groups, unpaired Student’s *t* test between two groups, and paired *t* test for the data between acquisition trial and playback trial in step-through test. Results were presented as means ± standard deviation (SD). *p* values less than 0.05 were considered to be statistically significant.

## 5. Conclusions

GL ameliorates cognitive impairment and AD pathology in SAMP8 mice by suppressing neuroinflammation. These effects were associated with inhibition of HMGB1, resulting in the inactivation of the NF-κB pathway; GL reduces inflammatory mediators and attenuates microglial activation, Aβ deposition, and tau phosphorylation. Concurrently, GL restores the levels of aging-related factors, including α-Klotho and IGF-1. Notably, 18α-GL may exhibit greater efficacy than the 18β isomer. These findings highlight GL, particularly 18α, as a promising therapeutic candidate targeting inflammation-driven mechanisms in AD.

## Data Availability

The original contributions presented in this study are included in the article. Further inquiries can be directed to the corresponding authors.

## Abbreviations

AD	Alzheimer’s disease
18α-GL	18α-glycyrrhizinic acid
18β-GL	18β-glycyrrhizinic acid
Akt	Protein kinase B
APP	Amyloid precursor protein
Aβ	Amyloid-β
BACE1	Beta-site amyloid precursor protein cleaving enzyme 1
BBB	Blood–brain barrier
2′,3′-cGAMP	2′,3′-cyclic guanosine monophosphate-adenosine monophosphate
cGAS	cyclic GMP-AMP synthase
dsDNA	Double-stranded DNA
GA	Glycyrrhetinic acid
GL	Glycyrrhizin
GSK3β	Glycogen synthase kinase 3 beta
HMGB1	High mobility group box 1
IGF-1	Insulin-like growth factor I
IHC	Immunohistochemistry
MCI	Mild cognitive impairment
mtDNA	Mitochondrial DNA
PI3K	Phosphatidylinositol 3-kinase
p-Tau	Phosphorylated tau
SAMP8	Senescence-accelerated mouse prone 8
SAMR1	Senescence-accelerated mouse resistant 1
STING	Stimulator of interferon gene

## References

[1] Shafi A., Akmal M., Sethi A., Chauhdary Z. (2026). A mechanistic insight of neuro-inflammation signaling pathways and implication in neurodegenerative disorders. Inflammopharmacology.

[2] Castner S.A., Gupta S., Wang D., Moreno A.J., Park C., Chen C., Poon Y., Groen A., Greenberg K., David N. (2023). Longevity factor klotho enhances cognition in aged nonhuman primates. Nat. Aging.

[3] López-Valdés H.E., Hernández-Lucas M., Rodríguez-Fabián G.D.J., Esteban-Román N.F., Gutiérrez-Juárez R., Arrieta-Cruz I., Martínez-Coria H. (2025). The Anti-Inflammatory Actions of Soluble Klotho in Brain Aging and Its Main Associated Diseases. Int. J. Mol. Sci..

[4] Kaur N., Aran K.R. (2025). Uncovering the intricacies of IGF-1 in Alzheimer’s disease: New insights from regulation to therapeutic targeting. Inflammopharmacology.

[5] Paudel Y.N., Angelopoulou E., Piperi C., Othman I., Aamir K., Shaikh M.F. (2020). Impact of HMGB1, RAGE, and TLR4 in Alzheimer’s Disease (AD): From Risk Factors to Therapeutic Targeting. Cells.

[6] Zhang Y., Zou M., Wu H., Zhu J., Jin T. (2024). The cGAS-STING pathway drives neuroinflammation and neurodegeneration via cellular and molecular mechanisms in neurodegenerative diseases. Neurobiol. Dis..

[7] Akiguchi I., Pallàs M., Budka H., Akiyama H., Ueno M., Han J., Yagi H., Nishikawa T., Chiba Y., Sugiyama H. (2017). SAMP8 mice as a neuropathological model of accelerated brain aging and dementia: Toshio Takeda’s legacy and future directions. Neuropathology.

[8] Cheng X.R., Zhou W.X., Zhang Y.X. (2014). The behavioral, pathological and therapeutic features of the senescence-accelerated mouse prone 8 strain as an Alzheimer’s disease animal model. Ageing Res. Rev..

[9] Amagaya S., Sugishita E., Ogihara Y., Ogawa S., Okada K., Aizawa T. (1984). Comparative studies of the stereoisomers of glycyrrhetinic acid on anti-inflammatory activities. J. Pharmacobio-Dyn..

[10] Papaevgeniou N., Sakellari M., Jha S., Tavernarakis N., Holmberg C.I., Gonos E.S., Chondrogianni N. (2016). 18α-Glycyrrhetinic Acid Proteasome Activator Decelerates Aging and Alzheimer’s Disease Progression in Caenorhabditis elegans and Neuronal Cultures. Antioxid. Redox Signal..

[11] Wagle A., Seong S.H., Zhao B.T., Woo M.H., Jung H.A., Choi J.S. (2018). Comparative study of selective in vitro and in silico BACE1 inhibitory potential of glycyrrhizin together with its metabolites, 18α- and 18β-glycyrrhetinic acid, isolated from Hizikia fusiformis. Arch. Pharm. Res..

[12] Ahmed S., Ma N., Kawanokuchi J., Matsuoka K., Oikawa S., Kobayashi H., Hiraku Y., Murata M. (2024). Taurine reduces microglia activation in the brain of aged senescence-accelerated mice by increasing the level of TREM2. Sci. Rep..

[13] Daneshgar N., Leidinger M.R., Le S., Hefti M., Prigione A., Dai D.F. (2022). Activated microglia and neuroinflammation as a pathogenic mechanism in Leigh syndrome. Front. Neurosci..

[14] Yaffe K., Lindquist K., Penninx B.W., Simonsick E.M., Pahor M., Kritchevsky S., Launer L., Kuller L., Rubin S., Harris T. (2003). Inflammatory markers and cognition in well-functioning African-American and white elders. Neurology.

[15] Festoff B.W., Sajja R.K., van Dreden P., Cucullo L. (2016). HMGB1 and thrombin mediate the blood-brain barrier dysfunction acting as biomarkers of neuroinflammation and progression to neurodegeneration in Alzheimer’s disease. J. Neuroinflamm..

[16] Lista S., Imbimbo B.P., Grasso M., Fidilio A., Emanuele E., Minoretti P., López-Ortiz S., Martín-Hernández J., Gabelle A., Caruso G. (2024). Tracking neuroinflammatory biomarkers in Alzheimer’s disease: A strategy for individualized therapeutic approaches?. J. Neuroinflamm..

[17] Feng W., Aikedan A., Sinha S.C., Gan L. (2026). Expanding roles of cGAS-STING signaling in neuroinflammation. J. Clin. Investig..

[18] Abraham C.R., Li A. (2022). Aging-suppressor Klotho: Prospects in diagnostics and therapeutics. Ageing Res. Rev..

[19] Ge S., Dong F., Tian C., Yang C.H., Liu M., Wei J. (2024). Serum soluble alpha-klotho klotho and cognitive functioning in older adults aged 60 and 79: An analysis of cross-sectional data of the National Health and Nutrition Examination Survey 2011 to 2014. BMC Geriatr..

[20] Cui L., Gao L., Geng H., Zhang H., Wei H. (2024). Analysis of the relationship between mild cognitive impairment and serum klotho protein and insulin-like growth factor-1 in the elderly. Technol. Health Care.

[21] Zhang L., Zheng L., Cheng Y. (2025). Down-Regulation of Klotho/FGF23 by Low-Expressed VEGFR2 Inhibits the GH/IGF-1/PI3K/AKT Signaling Pathway Inducing Intrauterine Growth Restriction in Rats. Am. J. Reprod. Immunol..

[22] Witkowska-Sędek E., Pyrżak B. (2020). Chronic inflammation and the growth hormone/insulin-like growth factor-1 axis. Cent. Eur. J. Immunol..

[23] Westwood A.J., Beiser A., Decarli C., Harris T.B., Chen T.C., He X.M., Roubenoff R., Pikula A., Au R., Braverman L.E. (2014). Insulin-like growth factor-1 and risk of Alzheimer dementia and brain atrophy. Neurology.

[24] Dunacka J., Grembecka B., Majkutewicz I., Wrona D. (2025). Central Insulin-like Growth Factor-1 Treatment Enhances Working and Reference Memory by Reducing Neuroinflammation and Amyloid Beta Deposition in a Rat Model of Sporadic Alzheimer’s Disease. Pharmaceuticals.

[25] Alqahtani S.M., Al-Kuraishy H.M., Al-Gareeb A.I., Albuhadily A.K., Shokr M.M., Alexiou A., Papadakis M., Batiha G.E. (2026). Pharmacological modulation of the PI3K/AKT/GSK3β axis: A new frontier in Alzheimer’s disease treatment. Inflammopharmacology.

[26] Pan J., Yao Q., Wang Y., Chang S., Li C., Wu Y., Shen J., Yang R. (2024). The role of PI3K signaling pathway in Alzheimer’s disease. Front. Aging Neurosci..

[27] Zaretski S., Nieto Torres J.L., Lei X., Nolan J.P., Hansen M., Adams P.D. (2025). Senescent cells secrete chromatin components via senescence-associated extracellular particles. bioRxiv.

[28] Alipour-Khezri E., Moqadami A., Zununi Vahed S., Barzegari A. (2025). Cytosolic DNA sensing pathway in senescence and aging: Underlying mechanisms and targeted interventions. Biomed. Pharmacother..

[29] Ding R., Li H., Liu Y., Ou W., Zhang X., Chai H., Huang X., Yang W., Wang Q. (2022). Activating cGAS-STING axis contributes to neuroinflammation in CVST mouse model and induces inflammasome activation and microglia pyroptosis. J. Neuroinflamm..

[30] Ferecskó A.S., Smallwood M.J., Moore A., Liddle C., Newcombe J., Holley J., Whatmore J., Gutowski N.J., Eggleton P. (2023). STING-Triggered CNS Inflammation in Human Neurodegenerative Diseases. Biomedicines.

[31] Chen Y., Li Q., Wang Z., Sun L.V., Hou S.X. (2025). A novel NFKB1 agonist remodels tumor microenvironment and activates dendritic cells to promote anti-tumor immunity in colorectal cancer. J. Transl. Med..

[32] de Mingo Pulido Á., Hänggi K., Celias D.P., Gardner A., Li J., Batista-Bittencourt B., Mohamed E., Trillo-Tinoco J., Osunmakinde O., Peña R. (2021). The inhibitory receptor TIM-3 limits activation of the cGAS-STING pathway in intra-tumoral dendritic cells by suppressing extracellular DNA uptake. Immunity.

[33] Liang Z.M., Lin L., Li S.R., Ma X.Y., Zhang W., Li N.N., Lai Z.H., Liang G., Zhao W., Zhao Y. (2026). cGAS-STING activation mediated by FAM83H-AS1/HMGB1 elicits NF-κB-dependent immune evasion in breast cancer. Sci. Bull..

[34] Ma H., Fang W., Li Q., Wang Y., Hou S.X. (2023). Arf1 Ablation in Colorectal Cancer Cells Activates a Super Signal Complex in DC to Enhance Anti-Tumor Immunity. Adv. Sci..

[35] Quintanilla R.A., Orellana D.I., González-Billault C., Maccioni R.B. (2004). Interleukin-6 induces Alzheimer-type phosphorylation of tau protein by deregulating the cdk5/p35 pathway. Exp. Cell Res..

[36] Morales I., Farías G., Maccioni R.B. (2010). Neuroimmunomodulation in the pathogenesis of Alzheimer’s disease. Neuroimmunomodulation.

[37] Kawanishi S., Wang G., Ma N., Murata M. (2025). Cancer Development and Progression Through a Vicious Cycle of DNA Damage and Inflammation. Int. J. Mol. Sci..

[38] Ikram F.Z., Arulsamy A., Retinasamy T., Shaikh M.F. (2022). The Role of High Mobility Group Box 1 (HMGB1) in Neurodegeneration: A Systematic Review. Curr. Neuropharmacol..

[39] Salminen A. (2026). Redox-sensitive high mobility group box 1 (HMGB1) protein is a multipotent regulator in the pathogenesis of Alzheimer’s disease. Neurochem. Int..

[40] Supasai S., Suntaratti P., Odton M., Longji T., Karananan T., Yasom S., Ampawong S., Limpanont Y., Mutirangura A. (2026). HMGB1 Box A gene therapy reverses cognitive and neuropathological features in AlCl_3_/D-galactose rat model of Alzheimer’s disease. Exp. Neurol..

[41] Govindarajulu M., Ramesh S., Beasley M., Lynn G., Wallace C., Labeau S., Pathak S., Nadar R., Moore T., Dhanasekaran M. (2023). Role of cGAS-Sting Signaling in Alzheimer’s Disease. Int. J. Mol. Sci..

[42] Quan S., Fu X., Cai H., Ren Z., Xu Y., Jia L. (2025). The neuroimmune nexus: Unraveling the role of the mtDNA-cGAS-STING signal pathway in Alzheimer’s disease. Mol. Neurodegener..

[43] Chami L., Buggia-Prévot V., Duplan E., Del Prete D., Chami M., Peyron J.F., Checler F. (2012). Nuclear factor-κB regulates βAPP and β- and γ-secretases differently at physiological and supraphysiological Aβ concentrations. J. Biol. Chem..

[44] Chen C.H., Zhou W., Liu S., Deng Y., Cai F., Tone M., Tone Y., Tong Y., Song W. (2012). Increased NF-κB signalling up-regulates BACE1 expression and its therapeutic potential in Alzheimer’s disease. Int. J. Neuropsychopharmacol..

[45] Gaikwad S., Puangmalai N., Bittar A., Montalbano M., Garcia S., McAllen S., Bhatt N., Sonawane M., Sengupta U., Kayed R. (2021). Tau oligomer induced HMGB1 release contributes to cellular senescence and neuropathology linked to Alzheimer’s disease and frontotemporal dementia. Cell Rep..

[46] Mary A., Mancuso R., Heneka M.T. (2024). Immune Activation in Alzheimer Disease. Annu. Rev. Immunol..

[47] Aranda-Abreu G.E., Rojas-Durán F., Hernández-Aguilar M.E., Herrera-Covarrubias D., Tlapa-Monge L.R., Mestizo-Gutiérrez S.L. (2026). Alzheimer’s Disease as a Disorder of Neuroimmune Dysregulation. Neurol. Int..

[48] Yang Y., Yang F., Yao M., Li L., Li H. (2026). Decoding the key mechanisms of ferroptosis and inflammation: Emerging therapeutic targets for Alzheimer’s disease. J. Alzheimers Dis..

[49] Yu J., Qu H., Xu W., Fang Y., Wang N., Shi W. (2026). Mechanisms and therapeutic potential of glycyrrhizic acid: Insights into key signaling pathways and disease modulation (Review). Mol. Med. Rep..

[50] Tabuchi M., Imamura S., Kawakami Z., Ikarashi Y., Kase Y. (2012). The blood-brain barrier permeability of 18β-glycyrrhetinic acid, a major metabolite of glycyrrhizin in Glycyrrhiza root, a constituent of the traditional Japanese medicine yokukansan. Cell. Mol. Neurobiol..

[51] Feng Y.L., Shui L., Jiang Y.X., Wang Y.N., Chen L., Chen H., Wang W.B. (2025). Licorice in nephropathy treatment: Phytochemical compositions and pharmacological mechanisms. Front. Pharmacol..

[52] Kao T.C., Shyu M.H., Yen G.C. (2010). Glycyrrhizic acid and 18beta-glycyrrhetinic acid inhibit inflammation via PI3K/Akt/GSK3beta signaling and glucocorticoid receptor activation. J. Agric. Food Chem..

[53] Amrutha S., Prasad T.S.K., Modi P.K. (2026). Glycyrrhizic Acid Attenuates Aβ(42)-Induced Neurodegeneration Through Coordinated Regulation of Oxidative Stress, Synaptic Markers, and Key Alzheimer’s Signaling Pathways. Cells.

[54] Classen-Houben D., Schuster D., Da Cunha T., Odermatt A., Wolber G., Jordis U., Kueenburg B. (2009). Selective inhibition of 11beta-hydroxysteroid dehydrogenase 1 by 18alpha-glycyrrhetinic acid but not 18beta-glycyrrhetinic acid. J. Steroid Biochem. Mol. Biol..

[55] Xu R., Xiao Q., Cao Y., Yang J. (2013). Comparison of the exposure of glycyrrhizin and its metabolites and the pseudoaldosteronism after intravenous administration of alpha- and beta-glycyrrhizin in rat. Drug Res..

[56] Aggarwal N.T., Mielke M.M. (2023). Sex Differences in Alzheimer’s Disease. Neurol. Clin..

[57] Li M., Peng S., Bu J., Quan S., Liu L., Yue Z., Wang L., Li Y. (2025). Glycyrrhizic acid alleviates gefitinib-induced liver injury by regulating the p53/p21 pathway and releasing cell cycle arrest. Food Chem. Toxicol..

[58] Hou S., Zhang T., Li Y., Guo F., Jin X. (2017). Glycyrrhizic Acid Prevents Diabetic Nephropathy by Activating AMPK/SIRT1/PGC-1α Signaling in db/db Mice. J. Diabetes Res..

[59] Pan Y.L. (2014). The effects of glycyrrhizin on acute pancreatitis in mice. Eur. Rev. Med. Pharmacol. Sci..

[60] Rossi T., Fano R.A., Castelli M., Malagoli M., Ruberto A.I., Baggio G., Zennaro R., Migaldi M., Barbolini G. (1999). Correlation between high intake of glycyrrhizin and myolysis of the papillary muscles: An experimental in vivo study. Pharmacol. Toxicol..

[61] Wang G., Hiramoto K., Ma N., Yoshikawa N., Ohnishi S., Murata M., Kawanishi S. (2021). Glycyrrhizin Attenuates Carcinogenesis by Inhibiting the Inflammatory Response in a Murine Model of Colorectal Cancer. Int. J. Mol. Sci..

